# Research trends of artificial intelligence in pancreatic cancer: a bibliometric analysis

**DOI:** 10.3389/fonc.2022.973999

**Published:** 2022-08-02

**Authors:** Hua Yin, Feixiong Zhang, Xiaoli Yang, Xiangkun Meng, Yu Miao, Muhammad Saad Noor Hussain, Li Yang, Zhaoshen Li

**Affiliations:** ^1^ Department of Gastroenterology, General Hospital of Ningxia Medical University, Yinchuan, China; ^2^ Postgraduate Training Base in Shanghai Gongli Hospital, Ningxia Medical University, Shanghai, China; ^3^ Department of Anesthesia, Dar Ul Shafa Hospital Sialkot, Punjab, Pakistan; ^4^ Clinical Medical College, Ningxia Medical University, Yinchuan, China

**Keywords:** Artificial intelligence, pancreatic cancer, AI, bibliometric, trends

## Abstract

**Purpose:**

We evaluated the related research on artificial intelligence (AI) in pancreatic cancer (PC) through bibliometrics analysis and explored the research hotspots and current status from 1997 to 2021.

**Methods:**

Publications related to AI in PC were retrieved from the Web of Science Core Collection (WoSCC) during 1997-2021. Bibliometrix package of R software 4.0.3 and VOSviewer were used to bibliometrics analysis.

**Results:**

A total of 587 publications in this field were retrieved from WoSCC database. After 2018, the number of publications grew rapidly. The United States and Johns Hopkins University were the most influential country and institution, respectively. A total of 2805 keywords were investigated, 81 of which appeared more than 10 times. Co-occurrence analysis categorized these keywords into five types of clusters: (1) AI in biology of PC, (2) AI in pathology and radiology of PC, (3) AI in the therapy of PC, (4) AI in risk assessment of PC and (5) AI in endoscopic ultrasonography (EUS) of PC. Trend topics and thematic maps show that keywords " diagnosis ", “survival”, “classification”, and “management” are the research hotspots in this field.

**Conclusion:**

The research related to AI in pancreatic cancer is still in the initial stage. Currently, AI is widely studied in biology, diagnosis, treatment, risk assessment, and EUS of pancreatic cancer. This bibliometrics study provided an insight into AI in PC research and helped researchers identify new research orientations.

## Introduction

Pancreatic cancer (PC) is a common cancer of the digestive system. It is known as the "king of cancers" because of its high mortality rate. According to the World Cancer Report 2020, the 1-year survival rate of pancreatic cancer after diagnosis is 24%, and the 5-year survival rate is only 9% (1). However, for those with early-stage cancer whose tumor was confined to the primary site, the 5-year relative survival rate increased to 39.4 percent (2). Improving survival in patients with pancreatic cancer may require in-depth research on multiple aspects, including early diagnosis, health management and treatment (3, 4). In this process, a large amount of biomedical data will be generated. How to efficiently organize, integrate, understand and analyze these big data to provide help for medical professionals is a current scientific problem, and the emergence of artificial intelligence has solved this difficulty well.

Artificial intelligence (AI) is a branch of computer science that employs computers to imitate human brain processes and intelligent behaviors like learning, reasoning, thinking, and planning (5). With the advancement of machine learning techniques, particularly deep learning and neural networks, AI has shown promise in the diagnosis, treatment, and prognosis of pancreatic cancer (6). Support vector machine (SVM) algorithm was used to analyze RNA-SEQ data to identify biomarkers for early diagnosis of pancreatic ductal adenocarcinoma (PDAC) (7). Endoscopic ultrasonography (EUS) and computerized tomography (CT) images were analyzed by artificial neural network (ANN), convolutional neural network (CNN), and other artificial intelligence techniques in imaging diagnosis, which can better predict the diagnosis of pancreatic cancer and effectively reduce the influence of experience and subjective factors on accuracy (8–10). Furthermore, an AI model based on preoperative CT was able to accurately predict the occurrence of postoperative pancreatic fistula (POPF) clinical complications following pancreaticoduodenectomy (PD) surgery, especially at the intermediate risk level (11). The AI system could help surgeons optimize their preoperative strategies. AI is rapidly changing the field of medicine, and academic research on this topic in pancreatic cancer had proliferated in recent years, which also indicates the need for a comprehensive analysis of research patterns and trends of AI in pancreatic cancer. The importance of summarizing global research trends and hot spots is critical, yet no bibliometric analysis study exists in this field.

Bibliometric analysis is an interdisciplinary subject that uses information visualization methods to make quantitative analysis and summary of the indicators of authors, journals, countries, institutions, references and keywords of worldwide literatures in a certain field (12). In this way, we can understand the knowledge structure more systematically and intuitively, and identify the frontier or hot spot in a certain research field (13). Therefore, this study aims to conduct a comprehensive analysis of the publication about AI in PC, and provide an overall view and direction for future work to promote AI in pancreatic cancer research.

## Materials and methods

### Database and search strategy

**Figure 1:**
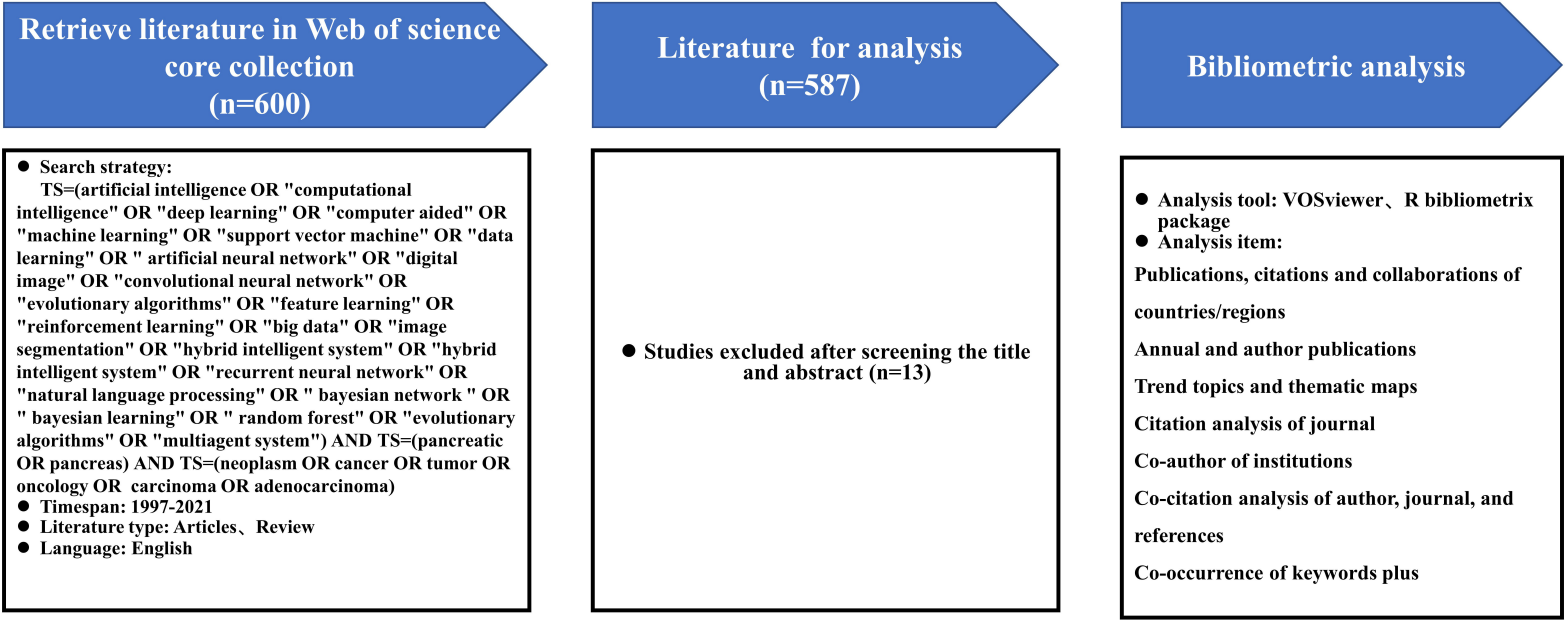
Procedures of bibliometric analysis: retrieval strategy, bibliometric analysis tools and items.

We used the Web of Science Core Collection (WoSCC) as our data source. Since WoSCC covers the most vital data sources for bibliometric analysis, it was the most frequently used and accepted database in scientific or bibliometric studies, with a more consistent and standardized record in multidisciplinary literature research (14). All data were retrieved from WoSCC on May 20, 2022 to avoid the bias that could occur due to continuous updating of the WoSCC database. The search strategy was described as follows: TS=(artificial intelligence OR "computational intelligence" OR "deep learning" OR "computer aided" OR "machine learning" OR "support vector machine" OR "data learning" OR " artificial neural network" OR "digital image" OR "convolutional neural network" OR "evolutionary algorithms" OR "feature learning" OR "reinforcement learning" OR "big data" OR "image segmentation" OR "hybrid intelligent system" OR "hybrid intelligent system" OR "recurrent neural network" OR "natural language processing" OR " bayesian network " OR " bayesian learning" OR " random forest" OR "evolutionary algorithms" OR "multiagent system") AND TS=(pancreatic OR pancreas) AND TS=(neoplasm OR cancer OR tumor OR oncology OR carcinoma OR adenocarcinoma). The document type was restricted to articles and reviews, timespan was set from 1997 to 2021, and the publications’ language was limited to English. In order to guarantee the representativeness of the included publications, the search results were filtered by title and abstract to exclude irrelevant articles. After 13 irrelevant literatures were excluded, 587 publications were finally extracted and exported in plain text format for analysis. The detailed retrieval process is shown in **Figure 1**.

### Statistical analysis

The bibliometrix package of R software 4.0.3 was used to analyze the collected data. Publications, citations and collaborations of countries/regions, annual publications, and author publications were analyzed and the bar chart of annual publications was derived using Microsoft Excel 2019. In addition, the Bibliometrix package was also used to generate trend topics and thematic maps. The quantity and quality of scientific researcher's academic production is evaluated by H-index that proposed by Hirsch (15). A scientist has index h if h of his or her the number of papers published over n years (Np) have at least h citations each and the other (Np – h) papers have ≤h citations each. VOSviewer (version 1.6.10) was used to visually analyze network maps of co-authors of institutions, co-citation of author/journal/references, citation of journal, and co-occurrence of keywords. Different clusters in the network map were represented by different colors and collaborations or co-citations were indicated by connection lines. The size of the circle indicates the number of documents, references, or keywords, while the thickness of the connection line indicates the strength of the link.

## Result

### Bibliometric analysis of annual publications

**Figure 2:**
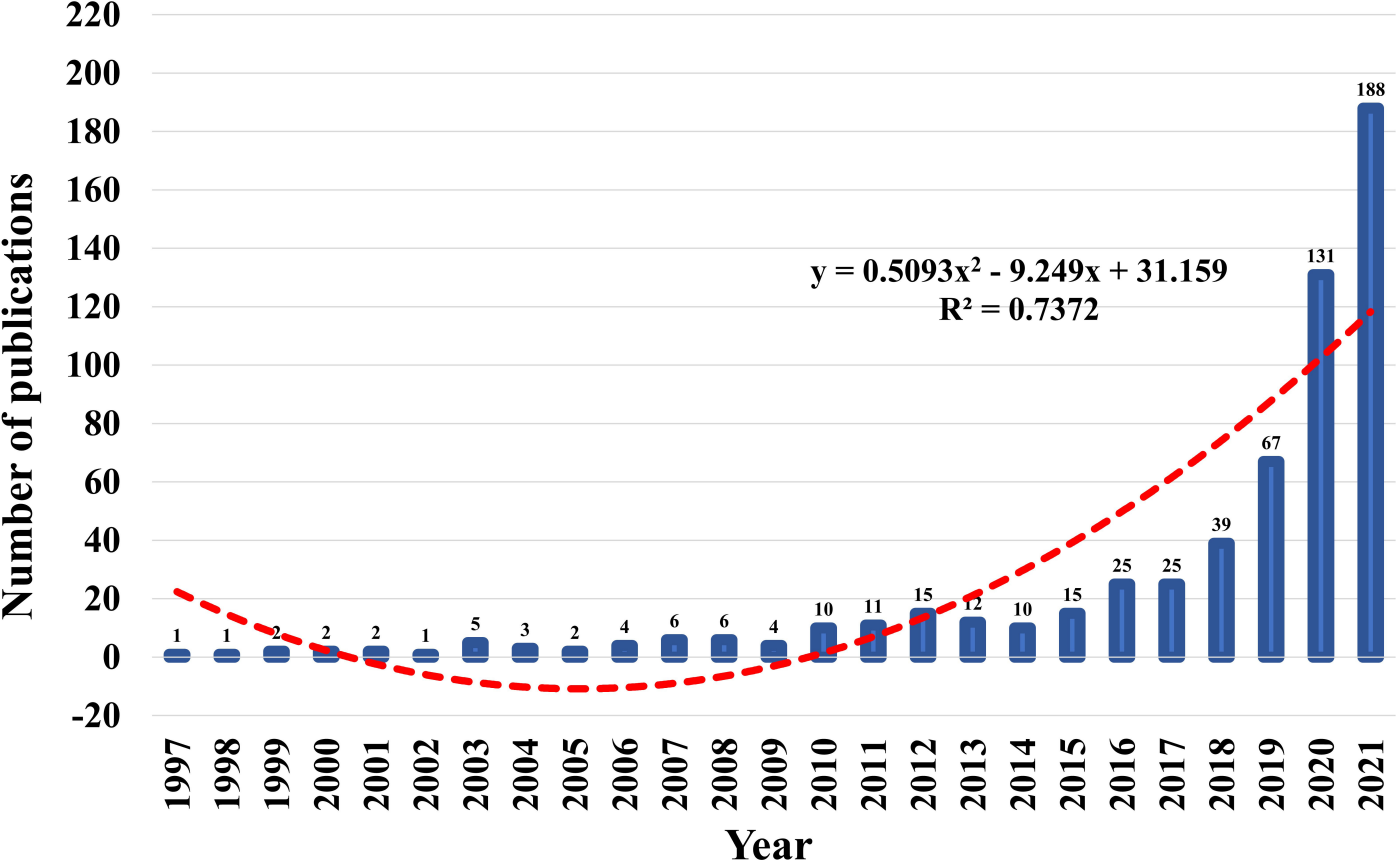
Trends of annual publications on research of AI in pancreatic cancer and the polynomial fitted curves (red dotted line).

Between 1997 and 2021, the WoSCC core database discovered 587 publications on the topic of AI in pancreatic cancer. **Figure 2** showed the trends of annual publications on research of AI in pancreatic cancer. Before 2017, the annual output of AI-related research on pancreatic cancer grew slowly, with only 25 publications published annually at most, indicating that it was in its infancy. But after 2018, the number of publications grew rapidly, reaching 188 in 2021, seven times that of 2017. In the past 4 years, the cumulative number of published documents accounted for 72.4% of all publications. A polynomial model fitting (R2=0.7372) predicted a significant correlation between publication year and publication output, and the number of literatures in this field will continue to increase in the future, indicating that the application of AI in pancreatic cancer has become one of the current frontier fields.

### The publishing performance of countries/regions

**Figure 3:**
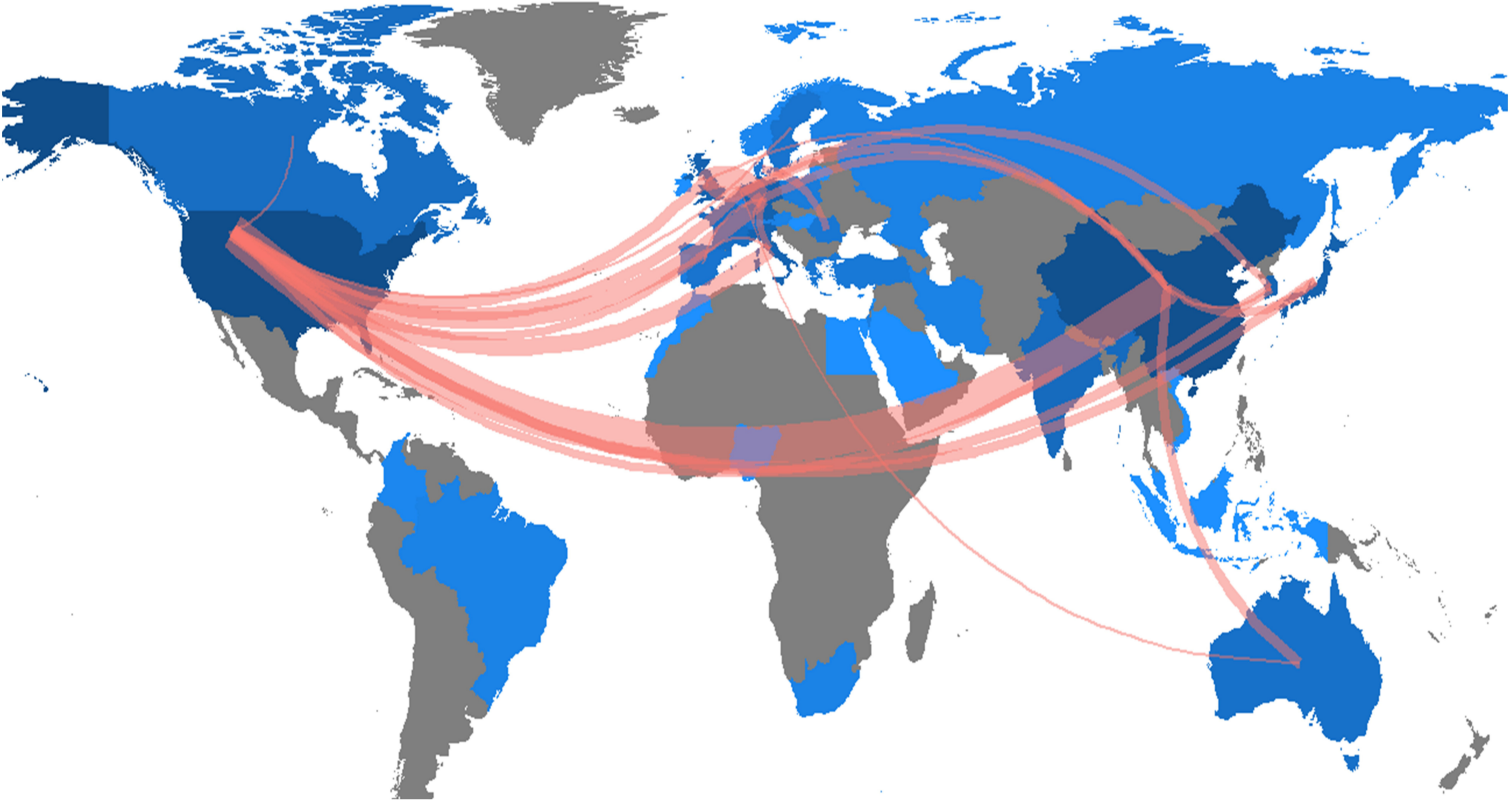
Country/region collaboration map. The Darker blues indicated higher collaboration rates, the wider the link line, the higher the rate of collaboration between the two countries. Countries with more than three shared papers were shown by connectors.

The research of AI in the field of PC was highlighted in 41countries across the world. The number of publications in each country was ranked to explore the global geographical distribution of publications in this field. In this study, it was found that the United States (181) was the most productive country, followed by China (154), Japan (33), Germany (29), and Korea (29), and the top 5 countries in total citations were the United States (4189), China (1785), Japan (596), Germany (466) and Italy (424). **Table 1** showed the 15 countries with the highest publication volume and total citations. **Figure 3** showed the country/region collaboration map worldwide. The Darker blues indicated higher collaboration rates. Countries that shared more than three papers were shown as link lines, and the wider the link line, the higher the rate of collaboration between the two countries. The highest number of collaborations was between the USA and China (30), followed by 12 and 8 collaborations between Germany and United Kingdom, and between USA and Germany, respectively. **Supplementary Table S1** listed top 10 collaborative countries/regions.

**Table 1:** Top 15 countries/regions with highest publications and total citations on AI in pancreatic cancer.

Rank	Country	Publications	Rank	Country	Total Citations
1	USA	181	1	USA	4189
2	CHINA	154	2	CHINA	1785
3	JAPAN	33	3	JAPAN	596
4	GERMANY	29	4	GERMANY	466
5	KOREA	29	5	ITALY	424
6	ITALY	24	6	ROMANIA	405
7	INDIA	14	7	KOREA	333
8	NETHERLANDS	14	8	FRANCE	295
9	UNITED KINGDOM	14	9	CANADA	237
10	FRANCE	11	10	AUSTRALIA	232
11	CANADA	10	11	UNITED KINGDOM	232
12	AUSTRALIA	8	12	NETHERLANDS	204
13	ROMANIA	7	13	SWEDEN	138
14	SWEDEN	6	14	SINGAPORE	135
15	SWITZERLAND	6	15	CYPRUS	66

### Bibliometric analysis of institutions

**Figure 4:**
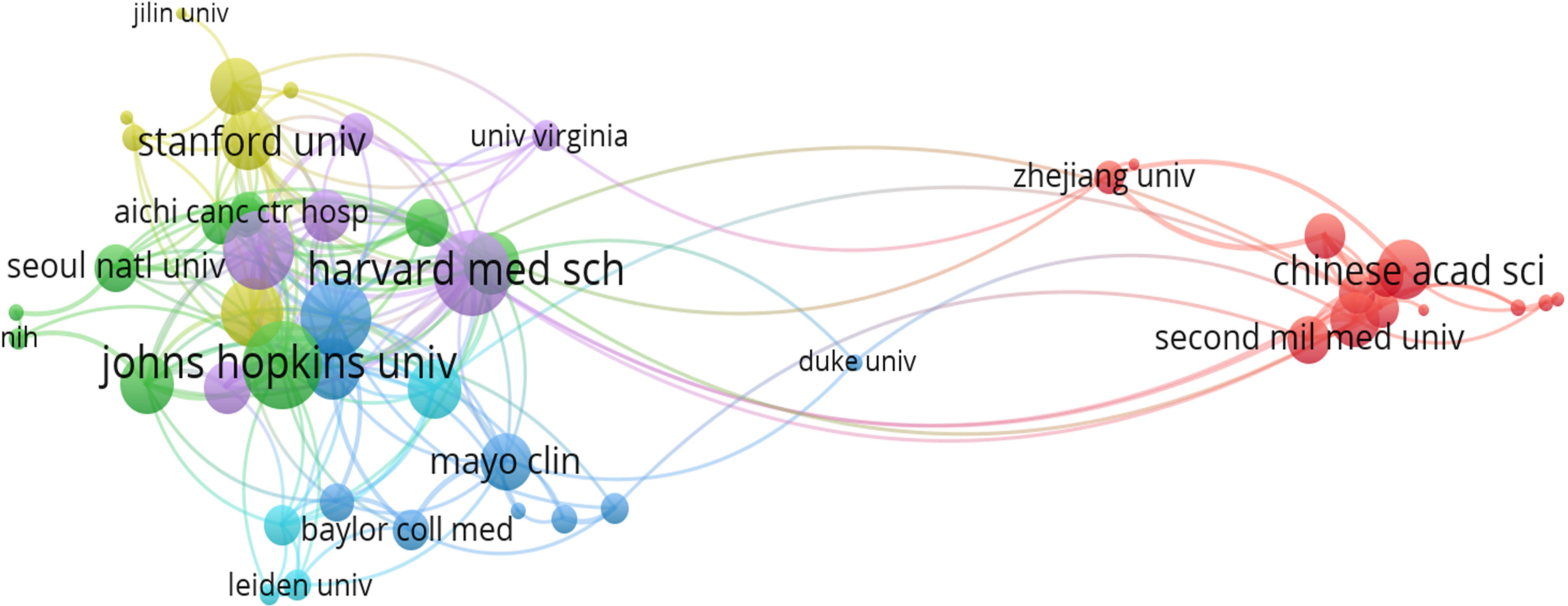
Analysis of co-authorship among institutions with more than five publications. Clusters were identified by color, the size of the circles represented the number of publications, and the thickness of the lines showed the strength of the association.

There were 1030 institutions from different countries involved in the publications of AI in the field of PC research. The Johns Hopkins University, with 64 studies analyzed by bibliometrix package, Zhe Jiang University (46), Fu Dan University (45), Seoul National University (39), and Memorial Sloan-Ketterlin Cancer Center (32), were the top five institutions. **Table 2** showed the top 15 institutions in terms of publications and citations. The co-authorship analysis showed that 51 institutions published more than five papers, and finally showed collaboration among 49 institutions after excluding two unrelated terms (**Figure 4**). In terms of linking strength, the leading five institutes included Johns Hopkins University (total link strength 34 times), Harvard Medical School (33), University of Pittsburgh (29), Memorial Sloan-Ketterlin Cancer Center (28), and The University of Texas MD Anderson Cancer Center (23).

**Table 2:** Top 15 publications and total citations institutions on research of AI in pancreatic cancer.

Rank	Affiliations	Publications	Affiliations	Citations
1	JOHNS HOPKINS UNIV	69	JOHNS HOPKINS UNIV	1034
2	ZHEJIANG UNIV	46	MEM SLOAN KETTERING CANC CTR	565
3	FUDAN UNIV	45	UNIV VIRGINIA	543
4	SEOUL NATL UNIV	39	UNIV CALIF SAN DIEGO	526
5	MEM SLOAN KETTERING CANC CTR	32	FUDAN UNIV	393
6	TECH UNIV MUNICH	31	NATL CANC CTR	346
7	UNIV TEXAS MD ANDERSON CANC CTR	29	LEIDEN UNIV	337
8	MAYO CLIN	25	UNIV AMSTERDAM	335
9	UNIV PENN	25	YALE UNIV	279
10	HARVARD MED SCH	23	STANFORD UNIV	268
11	SHANGHAI JIAO TONG UNIV	21	TECH UNIV MUNICH	264
12	SUN YAT SEN UNIV	21	UNIV TEXAS MD ANDERSON CANC CTR	248
13	YONSEI UNIV	20	UNIV PITTSBURGH	235
14	SICHUAN UNIV	19	SEOUL NATL UNIV	227
15	UNIV PITTSBURGH	19	MAYO CLIN	218

### Contributions of top journals and authors

**Figure 5:**
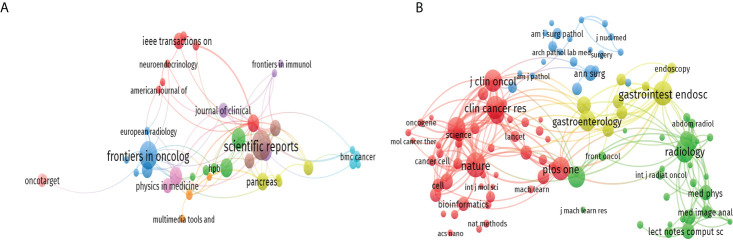
The citation and co-citation analysis of journal. **(A)** Network map of citation analysis of journals with more than three publications. **(B)** Network map of co-citation analysis of journals with more than fifty citations.

A total of 321 journals published research in this field, including 45 journals with more than three publications (**Figure 5A**). The top 10 journals contributed 114 publications, accounting for nearly 19.4% of the 587 publications retrieved. Scientific Reports published the most literature (20 publications, 3.4%), followed by Frontiers in Oncology (18 publications), Cancers (12 publications), Clinical Cancer Research (10 publications), and Medical Physics (10 publications). More details were shown in **Supplementary Table S2**. We analyzed all the journals and found that 104 of them had more than 50 citations (**Figure 5B**). The top ten co-cited journals that published related papers were included in **Supplementary Table S3**. The most co-cited journal was Nature (467 citations), followed by Gastrointest Endoscopy (453 citations), Plos One (424 citations), Radiology (417 citations), and Clinical Cancer Research (412 citations).

In this study, 3747 authors participated in AI-PC related research, and 27 authors among them published more than five research publications. **Table 3** listed the 10 most productive authors. Wang L, Li J, and Lu JP ranked in the top 3, with10, 9, and 9 articles, respectively. While author Hruban RH from Johns Hopkins University in the United States had the highest H-index. The H-index, proposed by Hirsch, combines the number of publications an author has published with the number of citations to better assess a scientist's impact.

**Table 3:** Top 10 productive authors on AI in pancreatic cancer.

Rank	Author	Publications	H-index	Total Citations
1	WANG L	10	5	82
2	LI J	9	4	61
3	LU JP	9	3	40
4	HRUBAN RH	7	6	282
5	JANG JY	7	5	208
6	KIM Y	7	4	76
7	PARK S	7	4	118
8	WANG Y	7	4	45
9	ZHANG H	7	3	47
10	ZHANG J	7	3	51

### Documents citation and references co-citation analyses

VOSviewer citation analysis showed that 44 of the 587 documents were cited more than 50 citations. The top three cited documents included “Molecular classification of human carcinomas by use of gene expression signatures” (520 citations) published in Cancer Research (16), followed by “Genome-wide cell-free DNA fragmentation in patients with cancer” (293 citations) published in Nature (17), and “Quantitative multiplex immunohistochemistry reveals myeloid-inflamed tumor-immune complexity associated with poor prognosis” (247 citations) published in Cell Reports (18). **Table 4** listed the top ten documents with the highest citations.

**Table 4:** Top ten documents with the highest citations on research of AI in pancreatic cancer.

Rank	Papers	Author	Year/Journal	Total Citations
1	Molecular classification of human carcinomas by use of gene expression signatures	Su et al. (16)	2001 CANCER RES	520
2	Genome-wide cell-free DNA fragmentation in patients with cancer	Cristiano et al. (17)	2019 NATURE	293
3	Quantitative Multiplex Immunohistochemistry Reveals Myeloid-Inflamed Tumor-Immune Complexity Associated with Poor Prognosis	Tsujikawa et al. (18)	2017 CELL REP	247
4	Objective quantification of the Ki67 proliferative index in neuroendocrine tumors of the gastroentero pancreatic system: a comparison of digital image analysis with manual methods	Tang et al. (19)	2012 AM J SURG PATHOL	186
5	An Automatic Learning-Based Framework for Robust Nucleus Segmentation	Xing et al. (20)	2016 IEEE T MED IMAGING	173
6	Neural network analysis of dynamic sequences of EUS elastography used for the differential diagnosis of chronic pancreatitis and pancreatic cancer	Săftoiu et al. (21)	2008 GASTROINTEST ENDOSC	154
7	Adjuvant treatments for resected pancreatic adenocarcinoma: a systematic review and network meta-analysis	Liao et al. (22)	2013 LANCET ONCOL	150
8	Fast and robust online adaptive planning in stereotactic MR-guided adaptive radiation therapy (SMART) for pancreatic cancer	Bohoudi et al. (23)	2017 RADIOTHER ONCOL	148
9	Application of Artificial Intelligence to Gastroenterology and Hepatology	Le berre et al. (24)	2020 GASTROENTEROLOGY	137
10	Mirna expression profiles identify drivers in colorectal and pancreatic cancers	Piepoli et al. (25)	2012 PLOS ONE	120

Co-citation references analysis evaluated the link between documents that were cited together by other articles. The top ten references with the most citations in this field were also provided in **Supplementary Table S4**. Gillies RJ (2016, Radiology; 37 citations) (26), Breiman L (2001, Mach Learn; 37 citations) (27), and Rahib L (2014, Cancer Research 35 citations) (28) were the three references with the most citations.

### Trends based on keywords co-occurrence analysis

**Figure 6:**
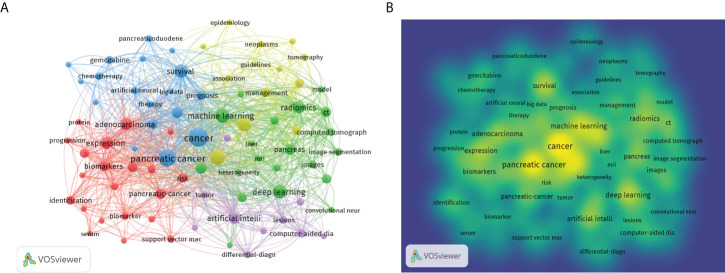
Bibliometric analysis of keywords by VOSviewer. **(A)** Map of Clusters based on keywords analysis. The size of the circle indicated occurrences of the keywords, different colors indicated the variety of clusters. **(B)** Keywords density visualization map. Different colors represented the frequency of keyword occurrences, and yellow indicated the highest frequency.

The purpose of keywords co-occurrence analysis is to look into the relationship between keywords in a set of publications in order to uncover hot themes and help scholars better grasp current scientific concerns. The co-occurrence keywords in the WoSCC database were analyzed using VOSviewer. A total of 2805 keywords were investigated, 81 of which appeared more than ten times. **Figure 6A** showed the visual network map of keywords co-occurrence. Nodes in different colors represented different types of clusters, node size represented the occurrence of keywords, and a thick connection line showed a close relationship between two items. The top three keywords were “cancer” with 132 occurrences, “pancreatic cancer” with 103 occurrences, and “diagnosis” with 80 occurrences. **Figure 6B** was a density visualization map based on the frequency of keyword occurrences. **Table 5** showed the number of occurrences, total link strength and theme description of each cluster. According to the results of cluster analysis, these keywords were divided into five clusters: cluster (1) AI in biology of pancreatic cancer; cluster (2) AI in pathology and radiology of pancreatic cancer; cluster (3) AI in therapy of pancreatic cancer; cluster (4) AI in risk assessment of pancreatic cancer; cluster (5) AI in endoscopic ultrasonography (EUS) of pancreatic cancer.

**Table 5:** Highly frequent major keywords in each cluster.

Cluster	Main keywords	Total link strength	Occurrences	Theme description
Cluster1 (red)	protein	33	10	AI in biology of pancreatic cancer
	expression	176	51	
	exosomes	37	10	
	biomarkers	116	31	
	validation	64	20	
	discovery	39	11	
	immunohistochemistry	43	12	
cluster2 (green)	computed tomography	103	21	AI in pathology and radiology of pancreatic cancer
	computed-tomography	73	15	
	ct	166	35	
	image segmentation	55	17	
	radiomics	215	47	
	prediction	120	27	
	texture analysis	117	23	
	pathology	41	11	
cluster3 (blue)	cancer	469	132	AI in therapy of pancreatic cancer
	chemotherapy	64	18	
	immunotherapy	41	10	
	prognosis	132	28	
	survival	282	59	
	therapy	74	16	
	resection	59	12	
cluster4 (yellow)	classification	260	65	AI in health management of pancreatic cancer
	diagnosis	309	80	
	epidemiology	40	11	
	management	119	28	
	prognostic-factors	39	11	
	features	133	26	
cluster5 (purple)	chronic pancreatitis	49	12	AI in EUS of pancreatic cancer
	computer-aided diagnosis	103	23	
	differential-diagnosis	87	15	
	endoscopic ultrasonography	40	10	
	endoscopic ultrasound	47	14	
	fine-needle-aspiration	48	17	
	lesions	81	18	
	pancreatic adenocarcinoma	55	15	

### Trend topics and thematic maps analysis

**Figure 7:**
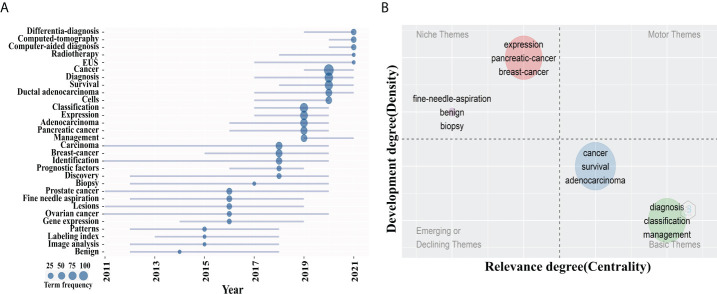
Theme analysis. **(A)** Trend topics map. The larger the circle is, the more occurrences keywords. **(B)** Thematic map of keywords plus. The x axis represented the centrality of a topic, which indicated the degree of interaction between network clusters in comparison to other clusters and provided information about its significance. The y axis showed the density that reflected the internal strength of a cluster network and may be considered as a measure of topic development. Keywords “ diagnosis “, “survival”, “classification”, and “management” in quadrant 4 (Basic themes).

Considering that the analysis results of different bibliometric tools may differ, we compared the co-occurrence keywords map of VOSviewer with the trend topics and thematic maps of Bibliomtrix software to identify research hotspots in this field. **Figure 7A** was the trend topics map generated by Bibliometrix package by analyzing the keywords plus. The larger the circle is, the more frequently keywords appear. It can be seen that the hot spots in 2019 were mainly “classification”, “management” and “expression”, corresponding to cluster 1 and 4, followed by “diagnosis” and “survival” in 2020, corresponding to cluster 3. In 2021, the major hotspots were “differential-diagnosis”, “computed tomography” and “EUS”, corresponding to clusters 2 and 5. The thematic map formed by two-dimensional matrix can better predict future research directions. The thematic map of keywords plus was shown in **Figure 7B**. The horizontal axis of a thematic map reflects centrality, whereas the vertical axis represents density. As a result, four quadrants are created: motor-themes, which are essential and well-developed in the first quadrant (top right corner); the second quadrant (top left corner): highly specialized/niche themes that have grown in popularity but are less relevant to the field; Emerging or declining themes, which indicate certain developing or fading themes, are shown in the third quadrant (lower left corner); Quadrant 4 (lower right corner): basic themes that are significant to the field but are underdeveloped. In the map, bubbles marked by the most frequently occurring keywords represent clusters, and the size of bubbles is proportional to the frequency of occurrence (29). It was worth noting that the two clusters located in quadrant 4, which keywords “diagnosis”, “survival”, “classification”, and “management” correspond to cluster 2, 3, 4 and 5 in the VOSviewer keyword network map, suggesting that they are the research frontiers in this field.

## Discussion

AI has permeated the medical field at an accelerated pace in the past few years, particularly in pancreatic cancer. This study used bibliometrix package of R software and VOSviewer visualization software to perform bibliometric analysis of AI applied to pancreatic cancer research in the past 25 years, providing a comprehensive perspective for the field. We objectively and systematically described the application status, development trend, and future research hotspots of AI in pancreatic cancer, so as to help scholars quickly understand the research status in this field and provide valuable directions for topic selection activities. The first part of this study analyzed publications trends, countries, institutions, authors, and journals. Then, cluster analysis was carried out on the keywords to identify the research hotspots in this field.

According to the results of publication trends, the number of AI in pancreatic cancer publications had increased rapidly over the past four years, accounting for 74.2% of the total number of papers published, but the annual number of AI in pancreatic cancer publications was still lower than in other diseases (30), which indicated that this field was in its infancy and more countries and researchers will be involved in research in the future.

The two nations with the most publications of AI in pancreatic cancer were the United States and China. Citation counts were a common indicator of professional recognition of scientific work so citation analysis was widely used to evaluate the quality of research work. The number of citations and country collaborations in the United States far outnumbers those in other countries, and the university with the most publications and citations was also based in the United States, demonstrating that the United States holds a significant position in this field and leads the world. Despite the fact that China had a large number of publications, the low citations show that China has to increase the quality and influence of its research. This may also be related to the late start of AI in pancreatic cancer research in China, resulting in low international academic influence. Wang L from China was the most published author with ten publications, while the author with the highest H-index was Hruban RH from Johns Hopkins University in the United States, who worked on AI in pathology and radiology of pancreatic cancer. The total number of papers published by each author in this field is not high, which may be related to the initial stage of AI in pancreatic cancer research.

In theme trend and thematic map, we found that the keywords “diagnosis”, “survival”, “classification”, and “management” may be hot research topics for the future. Keyword Co-occurrence Analysis classifies these keywords into five clusters, and we’ll discuss research hotspots and trends for each cluster individually.

Cluster 1: AI in biology of pancreatic cancer

The biomarker CA199 was most widely used in the detection of pancreatic cancer, but its specificity and sensitivity were relatively low (70% and 5%, respectively) (31, 32). In the past 20 years, sequencing technologies had made significant progress, AI massive sequencing data was used to establish statistical model, the genetic and epigenetic patterns associated with gene expression and gene product, calculation was complex and highly specific three-dimensional shape of the protein molecules, had become the realistic choice of biomarkers for early diagnosis of pancreatic cancer (7, 33). Stephen et al. established a machine learning model that integrated the fragmentation patterns of cell-free DNA across the genome with detection sensitivity ranging from 57% to more than 99%, specificity of 98%, and a total area of 0.94 under the curve value in seven cancer types including pancreatic cancer (17). This study ranked second in the top 10 citation publications and offers a proof-of-concept technique for human cancer screening, early diagnosis, and monitoring. In addition, miRNAs were used in a machine learning method to assist early diagnosis of PDAC (34).

Cluster 2: AI in pathology and radiology of pancreatic cancer

In the field of medical image analysis, deep learning has demonstrated a stable and exceptional performance. Artificial intelligence (AI) has been frequently utilized in CT and MRI imaging to identify pancreatic cancer and had showed notable benefits (35). Compared with radiologists, the convolutional neural network platform for CT-assisted diagnosis PC not only provided excellent diagnostic accuracy but also reduced the diagnosis time, indicating that AI has good clinical feasibility (10). MRI image segmentation by AI has been claimed to be more technically difficult than CT image segmentation (36). A convoluted neural network performed comparable to humans in recognizing the lesion, according to Liang et al (37). In hyperpolarized metabolic (HP)-magnetic resonance (MR) studies, AI was also employed to enhance the early detection of PDAC (38). To eliminate human bias in pathological specimen examination, AI approaches also performed well (39). The capacity of a multilayer perceptron neural network (MNN) to differentiate benign from malignant patients was developed and then assessed. In fact, the model had a 77 percent accuracy in classifying atypical fine-needle aspiration (FNA) biopsies as benign or malignant (40). Okon K et al. used neural network model to analyze the nuclear characteristics of patients undergoing surgery for pancreatic cancer, chronic pancreatitis and other tumors requiring pancreatic resection and the results showed that the correct rate of nuclear classification was 73% (41). Another study proposed a learning-based integrated framework for evaluating cell Ki-67 proliferation index, which had a higher accuracy than manual annotation and can provide indirect help for the diagnosis of pancreatic neuroendocrine tumors (42).

Cluster 3: AI in therapy of pancreatic cancer

The application of AI in the treatment of pancreatic cancer mainly focused on the prediction of pancreatic surgery risk, prognosis and chemotherapy response. AI can predict patient outcomes and postoperative complications by integrating multiple risk factors into the algorithm. Several researches applied an artificial intelligence system to create models and predict the occurrence of postoperative pancreatic fistula (POPF) following pancreaticoduodenectomy (11, 43, 44). The AI models established by Skawran et al. (45) and Kambkamba et al. (43) based on the imaging characteristics of preoperative CT and MRI respectively provided reliable predictability for the assessment of POPF. In assessing the prognosis of pancreatic cancer surgery, a subgroup of patients at low risk of mortality and morbidity in a study, Sahara et al. developed a predictive model based on machine learning to predict 30-day death following hepatopancreas surgery (46). The most important predictors of unexpected fatalities were age, sex, preoperative albumin level, preoperative platelet count, and disseminated malignancy. Palumbo et al. (47) used preoperative clinical and radiological data to build and verify a model for predicting early distant recurrence following upfront pancreaticoduodenectomy. This model proved to be useful in the treatment of pancreatic cancer patients. A study established a ML model to predict the risk of postoperative metastasis based on histological features of surgically removed pancreatic neuroendocrine tumor tissues (48). Zhang et al. developed a risk model using SVM, logistic regression, and lasso regression, which had a strong predictive effect on the likelihood of ICU admission and length of stay following PDAC surgery (49). In addition, one study presented a multiparameter analysis model to assess the predictive value of existing parameters and predicted long-term survival in patients with PDAC (50). AI also showed excellent performance in predicting chemotherapy response. For patients with pancreatic adenocarcinoma, a deep learning model can predict histological tumor response to neoadjuvant treatment, and the model was enhanced by include reductions in serum CA199 (51). Furthermore, the ANN dosage model was equivalent to the dose predicted by experts for pancreatic cancer stereotactic body radiation treatment (SBRT) (52). The use of artificial intelligence in the treatment of pancreatic cancer has shown to be promising. In the near future, Machine learning will play a greater role in pancreatic cancer treatment in the near future, thanks to thorough analyses of clinical factors, less-invasive biological samples, and radiological For patients with pancreatic adenocarcinoma, a deep learning model can predict histological tumor response to neoadjuvant treatment, and the model was enhanced by include reductions in serum CA199. Furthermore, the ANN dosage model was equivalent to the dose predicted by experts for pancreatic cancer stereotactic body radiation treatment (SBRT). The use of artificial intelligence in the treatment of pancreatic cancer has shown to be promising. Machine learning will play a greater role in pancreatic cancer treatment in the near future, thanks to thorough analyses of clinical factors, less-invasive biological samples, and radiological characteristics.

Cluster 4: AI in risk assessment of pancreatic cancer

Only a small percentage of patients (<15%) with pancreatic cancer had surgically resectable state after diagnosis (53), so identifying individuals at high risk for PDAC or with early stage of resectable pancreatic cancer was critical to prognosis. The use of AI-based risk assessment models to pancreatic cancer is gaining traction. Appelbaum et al. used diagnostic codes collected from electronic health record (EHR) data to design and verify a prediction model that identified persons at high risk for PDAC up to a year before diagnosis (54). They also attempted to improve the performance of the model using independent multicenter datasets and additional laboratory test features. AI-based PC risk prediction models were established by analyzing patients with chronic pancreatitis of focal mass lesions and patients with prediabetes and new-onset diabetes, respectively (55, 56). The specificity of these models in identifying PC was up to 94.0%. PCLs (pancreatic cystic lesions) were well-known pancreatic cancer precancerous symptoms. To find characteristics that can be utilized as imaging biomarkers to detect high-risk PCLs, researchers applied quantitative image analysis mixed with machine learning and artificial intelligence (AI) technologies (57–60). This cost-effective method will aid in the differentiation of benign and malignant PCL, as well as advise therapeutic decisions for better utilization of medical resources.

Cluster 5: AI in EUS of pancreatic cancer

With promising results, AI has been applied to help endoscopists in the EUS assessment of pancreatic lesions (61–63). Zhu et al. collected EUS image characteristics from patients with PC and chronic pancreatitis and chose 16 features for SVM classification, with a sensitivity rate of 94% (8). For neural network analysis, Săftoiu employed dynamic sequences of EUS elastography and contrast-enhanced EUS recordings (21, 64). When CP and PC were differentiated, superior diagnostic results were obtained, according to their findings. AI was also employed to discriminate between benign and malignant cells during EUS-guided pancreatic fine-needle aspiration. Multilayer perceptron neural networks (MNNs) trained on segment pictures of cell clusters collected from FNA were as accurate as the cytologist who first inspected the section, according to one research (40). This model may be widely used as a screening tool for pancreatic FNA specimens. In the future, as clinical decision-making becomes increasingly complex, AI will play an important role in assisting clinicians in handling large amounts of EUS data. For EUS training and quality control, Zhang et al. used AI to construct a station classification model and segmentation model that included a pancreas, abdominal aorta and portal confluence. EUS’s learning curve was reduced and quality control was enhanced because to this intelligence system (65).

## Conclusion

The research of artificial intelligence in pancreatic cancer is still in its initial stages. AI is now being researched in pancreatic cancer biology, diagnosis, therapy, risk assessment, and EUS. The application of artificial intelligence effectively avoids human error and improves work efficiency. This bibliometrics study provides an insight into AI in PC research, concentrating on the present condition of the discipline. This viewpoint aids researchers in recognizing the field’s hotspots and frontiers, as well as new research directions.

## Data availability statement

The original contributions presented in the study are included in the article/**Supplementary Material**. Further inquiries can be directed to the corresponding authors.

## Author contributions

The data was gathered by XM and YM. HY, FZ and XY analyzed the data and wrote the manuscript. MNH revised the language of the manuscript. ZL and LY conceived the study and reviewed the manuscript. All authors listed have made a substantial, direct, and intellectual contribution to the work and approved it for publication.

## Funding

This research was supported by University-level project grant (Grant Number XM2019094).

## Conflict of interest

The authors declare that the research was conducted in the absence of any commercial or financial relationships that could be construed as a potential conflict of interest.

## Publisher’s note

All claims expressed in this article are solely those of the authors and do not necessarily represent those of their affiliated organizations, or those of the publisher, the editors and the reviewers. Any product that may be evaluated in this article, or claim that may be made by its manufacturer, is not guaranteed or endorsed by the publisher.
