# Supplementary material for: Research trends of artificial intelligence in pancreatic cancer: a bibliometric analysis
Source: Front Oncol. 2022 Aug 2;12:973999. doi: 10.3389/fonc.2022.973999 (PMC9380440; doi:10.3389/fonc.2022.973999)
Supplement: Supplementary file 1 [file Table_1.docx]

Table S1 Top 10 collaborations countries

| **Rank** | **From** | **To** | **Frequency** |
| --- | --- | --- | --- |
| 1 | USA | CHINA | 33 |
| 2 | GERMANY | UNITED KINGDOM | 12 |
| 3 | USA | GERMANY | 8 |
| 4 | USA | ITALY | 8 |
| 5 | UNITED KINGDOM | NETHERLANDS | 7 |
| 6 | USA | NETHERLANDS | 6 |
| 7 | USA | UNITED KINGDOM | 6 |
| 8 | CHINA | AUSTRALIA | 5 |
| 9 | USA | JAPAN | 5 |
| 10 | USA | KOREA | 5 |

Table S2 Top ten co-citation analysis of cited source on AI in pancreatic cancer

| **Rank** | **Source** | **Co-Citations** | **Total link strength** |
| --- | --- | --- | --- |
| 1 | NATURE | 467 | 21215 |
| 2 | GASTROINTEST ENDOSC | 453 | 16812 |
| 3 | PLOS ONE | 424 | 17092 |
| 4 | RADIOLOGY | 417 | 14941 |
| 5 | CLIN CANCER RES | 412 | 19777 |
| 6 | CANCER RES | 397 | 19114 |
| 7 | J CLIN ONCOL | 390 | 16503 |
| 8 | SCI REP-UK | 366 | 13958 |
| 9 | GASTROENTEROLOGY | 336 | 14991 |
| 10 | NEW ENGL J MED | 294 | 12634 |

Table S3 Top ten journal with highest publications on AI in pancreatic cancer

| **Rank** | **Source** | **Documents** | **TC** | **IF** | **JCR** | **TLS** |
| --- | --- | --- | --- | --- | --- | --- |
| 1 | SCIENTIFIC REPORTS | 20 | 197 | 4.379 | Q1 | 5 |
| 2 | FRONTIERS IN ONCOLOGY | 18 | 99 | 6.244 | Q2 | 22 |
| 3 | CANCERS | 12 | 56 | 6.639 | Q2 | 14 |
| 4 | CLINICAL CANCER RESEARCH | 10 | 255 | 12.531 | Q1 | 3 |
| 5 | MEDICAL PHYSICS | 10 | 144 | 4.071 | Q2 | 14 |
| 6 | PLOS ONE | 10 | 219 | 3.240 | Q2 | 24 |
| 7 | PANCREAS | 9 | 65 | 3.327 | Q3 | 12 |
| 8 | WORLD JOURNAL OF GASTROENTEROLOGY | 9 | 110 | 5.742 | Q2 | 58 |
| 9 | ABDOMINAL RADIOLOGY | 8 | 40 | 3.039 | Q3 | 10 |
| 10 | GASTROINTESTINAL ENDOSCOPY | 8 | 368 | 9.427 | Q1 | 38 |

TC: total citation; IF: impact factor; JCR: journal citation reports；TLS: total link strength;

| **Author** | **Year** | **Journal** | **DOI** | **TC** | **TLS** |
| --- | --- | --- | --- | --- | --- |
| Gillies RJ | 2016 | RADIOLOGY | 10.1148/radiol.2015151169 | 37 | 157 |
| Breiman L | 2001 | MACH LEARN | 10.1023/a:1010933404324 | 37 | 73 |
| Rahib L | 2014 | CANCER RES | 10.1158/0008-5472.can-14-0155 | 35 | 83 |
| Siegel RL | 2019 | CA-CANCER J CLIN | 10.3322/caac.21551 | 31 | 77 |
| Ronneberger O | 2015 | LECT NOTES COMPUT SC | 10.1007/978-3-319-24574-4_28 | 31 | 50 |
| Bray F | 2018 | CA-CANCER J CLIN | 10.3322/caac.21492 | 30 | 73 |
| Eilaghi A | 2017 | BMC MED IMAGING | 10.1186/s12880-017-0209-5 | 29 | 122 |
| Aerts HJWL | 2014 | NAT COMMUN | 10.1038/ncomms5006 | 25 | 121 |
| Lecun Y | 2015 | NATURE | 10.1038/nature14539 | 25 | 62 |
| Kumar V | 2012 | MAGN RESON IMAGING | 10.1016/j.mri.2012.06.010 | 22 | 104 |

Table S4 Top ten cited reference on AI in pancreatic cancer

TC: total citation; IF: impact factor; JCR: journal citation reports；TLS: total link strength;
